# Oxidative DNA Damage Is Associated with Immune Remodeling and Therapeutic Response in High-Grade Serous Ovarian Cancer

**DOI:** 10.3390/cancers18152437

**Published:** 2026-07-29

**Authors:** Carson C. Edwards, Jenna M. Hedlich-Dwyer, Jianqing Zhang, Valeria L. Dal Zotto, Dongquan Chen, Rebecca C. Arend, Natalie R. Gassman

**Affiliations:** 1 Department of Obstetrics and Gynecology, Division of Gynecologic Oncology, Heersink School of Medicine, University of Alabama at Birmingham, Birmingham, AL 35294, USA; carsoncedwards@uabmc.edu (C.C.E.); rarend@uabmc.edu (R.C.A.); 2 Department of Pathology, Heersink School of Medicine, University of Alabama at Birmingham, Birmingham, AL 35294, USA; jhedlich@hfsctx.gov (J.M.H.-D.); vldalzotto@uabmc.edu (V.L.D.Z.); 3 Department of Radiation Oncology, Heersink School of Medicine, University of Alabama at Birmingham, Birmingham, AL 35294, USA; jianqingzhang@uabmc.edu; 4 Department of Medicine, Division of General Internal Medicine & Population Science, Heersink School of Medicine, University of Alabama at Birmingham, Birmingham, AL 35294, USA; dchen@uabmc.edu; 5 O’Neal Comprehensive Cancer Center, University of Alabama at Birmingham, Birmingham, AL 35294, USA

**Keywords:** ovarian cancer, DNA damage, oxidative stress, adaptive immunity, tumor–immune microenvironment, neoadjuvant chemotherapy

## Abstract

**Background/Objectives**: High-grade serous ovarian cancer (HGSOC) shows variable response to platinum-based neoadjuvant chemotherapy (NACT), with outcomes strongly linked to upfront treatment sensitivity. We evaluated whether oxidative DNA damage in pathologic samples is associated with improved clinical outcomes and reflects tumor–immune interactions. **Methods**: We analyzed matched pre- and post-NACT tumors from patients with stage III–IV HGSOC using Repair Assisted Damage Detection (RADD) to quantify total and oxidative DNA lesions (oxRADD). Gene expression profiling was performed on a subset of tumors using the NanoString PanCancer I/O 360. Associations with homologous recombination status, platinum sensitivity, recurrence, and survival were assessed. **Results**: Higher pre-NACT oxidative DNA damage was observed in tumors from patients who later recurred. Among recurrent tumors, elevated oxidative lesions were associated with improved overall survival (61.8 vs. 35.0 months; HR = 0.42, *p* = 0.037). Oxidative damage predicted recurrence (AUC = 0.71), supporting its utility in risk stratification. Tumors with serious oxidative damage showed reduced IDO1 and TGFβ signaling signatures, along with decreased B cell- and T cell-associated TIGIT signatures after NACT. **Conclusions**: These findings identify oxidative DNA damage as a potential pretreatment biomarker associated with recurrence, survival, and tumor–immune state, supporting its potential to impact therapeutic decision-making in HGSOC.

## Simple Summary

Ovarian cancer shows variable responses to platinum-based chemotherapies. There is a need for biomarkers that can predict therapeutic response to improve patient outcomes. We examined whether DNA damage levels, particularly oxidative DNA damage, could act as a biomarker of response. Using a novel DNA damage detection method, we demonstrated that oxidative DNA damage was predictive for cancer recurrence and homologous recombination repair competency. We also showed that DNA damage levels are associated with changes in the tumor–immune microenvironment. High oxidative DNA damage tumors showed reduced IDO1 and TGFβ signaling signatures. These findings link DNA damage to the tumor microenvironment and may identify oxidative DNA damage as a potential biomarker for recurrence, homologous recombination proficiency, survival, and tumor–immune state.

## 1. Introduction

High-grade serous ovarian cancer (HGSOC) is responsible for more deaths than any other gynecologic cancer in the United States [1]. Outcomes remain particularly poor for patients with advanced-stage disease, for whom five-year survival is near 30% despite improvements in operative management and systemic therapy options. High mortality for ovarian cancer patients is driven in large part by the development of platinum resistance and frequent recurrence within the first two years after therapy [2]. For late-stage patients who are unlikely to undergo successful primary cytoreductive surgery, neoadjuvant chemotherapy (NACT) followed by interval debulking surgery (IDS) has become an important treatment strategy that can improve the likelihood of achieving optimal cytoreduction [3]. While the surgical benefit of NACT is clear, cytotoxic treatment also reshapes the tumor microenvironment (TIME) through complex transcriptomic and immunologic adaptations, which have not yet been fully defined [4].

Recent investigations have demonstrated that NACT exposure triggers substantial remodeling of the ovarian TIME, including increased tumor-infiltrating lymphocytes alongside upregulation of immunosuppressive pathways. James et al. identified activating transcription factor 3 (ATF3) and early growth response 1 (EGR1) as master transcriptional regulators upregulated following NACT, coordinating stress-response programs that encompass both pro-tumorigenic signaling and immune regulatory mechanisms [4]. Importantly, post-NACT immune infiltrate and pathway signatures demonstrated prognostic associations with platinum-free interval (PFI), while pre-NACT features showed minimal correlation, suggesting that chemotherapy-induced TIME adaptations critically influence subsequent treatment response [4].

While retrospective analysis of these changes is informative, biomarker-driven prediction models for platinum response, treatment-related post-NACT remodeling, and secondary and tertiary therapeutic selection are needed. One potential tool for predicting stress responses and TIME changes may be genomic instability. DNA damage response and repair pathways are stimulated by stress response factors and oncogenic drivers, which alter DNA repair fidelity [5]. Furthermore, there has been increasing interest in the impact genomic stability has on shaping TIME, immune regulatory mechanisms, and ultimately treatment response [6].

Genomic instability is a hallmark of HGSOC, with approximately 50% of tumors exhibiting homologous recombination deficiency (HRD) due to *BRCA1/2* mutations or other DNA repair pathway alterations [7]. While HRD tumors demonstrate enhanced platinum sensitivity, homologous recombination proficient (HRP) tumors comprise a substantial fraction of HGSOC cases with limited therapeutic options beyond conventional chemotherapy [8]. Emerging evidence suggests that persistent DNA damage, particularly oxidative lesions, may influence tumor immunogenicity and immune checkpoint expression, potentially creating vulnerabilities amenable to immunotherapeutic intervention [9].

The current challenge is how to measure persistent DNA damage, specifically oxidative lesions, within patient samples to quantify genomic instability and assess it as a potential predictive biomarker for treatment response. Repair Assisted Damage Detection (RADD) enables direct quantification of DNA lesions within formalin-fixed paraffin-embedded (FFPE) tissues by employing DNA repair enzymes to detect and remove the lesions, followed by site-specific labeling of the damage site within the genome [10]. We previously employed RADD to assess the lesion composition within histological subtypes of ovarian cancers and determined that HGSOC showed elevated levels of oxidative lesions, which also correlated with the expression of PD-L1 [9].

More recently, we applied the broad-spectrum RADD, which detects oxidative, crosslinked, and alkylating lesions, along with uracil and abasic sites, to determine if DNA damage levels were predictive for response to Vigil immunotherapy, an autologous tumor cell vaccine, which significantly improved survival in HRP HGSOC patients receiving Vigil as maintenance therapy [11]. DNA damage levels measured by RADD predicted enhanced Vigil response, with high RADD scores associated with improved recurrent-free survival. These findings suggest that DNA damage-mediated alterations in the immune microenvironment may contribute to therapeutic response.

In this study, we used a RADD enzyme cocktail specific for oxidative lesions (oxRADD) to investigate the relationship between oxidative DNA damage, the immune microenvironment, and clinical outcomes in matched pre- and post-NACT HGSOC tumors. We hypothesized that quantification of oxidative lesions would reveal distinct immune phenotypes associated with platinum sensitivity and survival, providing mechanistic insights into chemotherapy-induced TIME adaptations to inform biomarker selection and treatment response.

## 2. Materials and Methods

### 2.1. Patient Cohort and Sample Collection

This study was approved by the University of Alabama at Birmingham Institutional Review Board (IRB-300008032 approved 30 September 2021, IRB-300008039 approved 22 May 2025, IRB-131007005 approved 1 December 2017). Written informed consent was obtained from all participants prior to specimen collection and clinical data extraction. Formalin-fixed paraffin-embedded (FFPE) tumor specimens were obtained from 40 patients who were initially classified as stage III–IV high-grade serous ovarian cancer and who received neoadjuvant chemotherapy (NACT) consisting of carboplatin (AUC 5–6) and paclitaxel (175 mg/m^2^) for 3–6 cycles between 2015 and 2022 (Supplementary Materials). Matched pre-NACT biopsies and post-NACT interval debulking specimens were required for inclusion. Clinical data, including age, FIGO stage, treatment regimen, platinum sensitivity (defined as platinum-free interval (PFI) ≥ 6 months), recurrence status, and survival outcomes, were extracted from electronic medical records with a minimum 12-month follow-up.

### 2.2. Homologous Recombination Status Determination

Homologous recombination status (homologous recombination proficient (HRP) or homologous recombination deficient (HRD)) was determined for all patients using Caris Life Science’s MI profiling or Foundation One CDx assay, plus germline testing. Patients were considered HRD if Foundation One CDx testing demonstrated loss of heterozygosity > 16%, read-out from Caris testing showed HRD positive, or if BRCA1/2 mutation was present on germline testing. Due to the heterogeneous timing and location of genetic testing, they were not completed by a single company, but the status recording in the chart was consistent.

### 2.3. Repair Assisted Damage Detection (RADD) Assay

The RADD assay was performed as previously described in [9,11,12] with the same controls and variability measures employed as in [11,12]. Briefly, FFPE sections were deparaffinized and sequentially rehydrated. Antigen retrieval using 10 mM sodium citrate pH 6.0 (Sigma-Aldrich, St. Louis, MO, USA, W3022600-1KG-K) with 0.05% Tween 20 (Fisher Scientific, Waltham, MA, USA, BP337-100) and 1 mM EDTA (Thermo Fisher, Waltham, MA, USA, AM9260G) was followed by heating using a microwave. For broad-spectrum DNA damage detection (Full RADD), uracil-DNA Glycosylase (UDG; New England Biolabs (NEB), Ipswich, MA, USA, M0280), formamidopyrimidine DNA glycosylase (FPG; NEB M0240), pyrimidine dimer glycosylase (T4PDG; NEB M0308), alkyladenine DNA glycosylase (AAG; NEB M0313), endonuclease VIII (EndoVIII; NEB M0299), and endonuclease IV (EndoIV; NEB M0304) in ThermoPol reaction buffer (20 mM Tris-HCl pH 8.0, 1 mM EDTA, 100 mM NaCl; NEB 9004) were added to the slides and incubated for 1 h at 37 °C. To assess for oxidative lesions specifically (oxRADD), a cocktail of FPG, EndoVIII, and Endo IV in ThermoPol reaction buffer was added to separate slides and incubated for 1 h at 37 °C. These enzymes specifically recognize and excise oxidative DNA lesions including 8-oxo-7,8-dihydroguanine, thymine glycol, formamidopyrimidines, and abasic sites, generating DNA strand breaks that are specific to oxidative DNA damage. A gap-filling solution of Klenow exo^-^ (Thermo Fisher, Waltham, MA, USA, EP0422) and digoxigenin-dUTP (Sigma-Aldrich, 11093088910) was added directly to the lesion removal solution, and the slides with the combination solution were incubated for an additional 1 h at 37 °C. Slides were washed in Tris-buffered saline (TBS, VWR Life Sciences, Radnor, PA, USA) with 0.1% Tween 20 (TBST) solution. Next, slides were washed and blocked using 5% goat serum (Thermo Fisher, PI31873) in phosphate-buffered saline (PBS) (Mediatech Inc, Manassas, VA, USA, 21-040-CV) for 30 min at room temperature (~22 °C). Then, slides were incubated overnight (~12 h) at 4 °C with anti-digoxigenin (Dig) antibodies (Abcam, Waltham, MA, USA, ab420) diluted to 1:250 in 5% goat serum. After incubation, the slides were washed, and Alexa Fluor 647 goat anti-mouse secondary (Life Technologies, Carlsbad, CA, USA, A21235) was incubated at a dilution of 1:400 in 5% goat serum in PBS for 1 h at room temperature. Hoechst 33342 (Life Technologies, PI62249) was added at a final dilution of 1:800 for 15 min at RT to stain the nuclei. Slides were then washed in PBS, dried, and mounted using ProLong Gold Antifade reagent (Life Technologies, Carlsbad, CA, USA, P36930). Slides were allowed to dry for 24 h at room temperature with minimal light exposure.

Imaging was performed using a Keyence (Osaka, Japan, BZ-X800) with a 10× objective (NA 0.45). Image acquisition settings were identical across all samples for direct tissue comparisons and analysis. An adjacent corresponding hematoxylin and eosin (H&E) stained section for each tumor was reviewed by a gynecologic pathologist to confirm the presence of tumor, annotate the tumor area, estimate the percentage of tumor, and note areas of necrosis, if any, within each tissue section. Imaging was performed on the tumor-containing tissues with each section at 960 × 720 resolution. To cover all of the tumor area within a section, multiple sections were stitched together. The number of sections ranged from 100 to 800. The intensity of each stitched image is then recorded and averaged across all stitches to yield the mean fluorescent intensity RADD score. Saturated pixels were removed with a binary threshold. Mean fluorescent intensity RADD scores for the isotype control Ig samples were <10^5^, demonstrating the specificity of the measured fluorescence intensity for the specific antibody used.

RADD fluorescence was quantified across the tumor-containing tissue section, including the tumor and the immediately adjacent stromal and peritumoral compartments. This approach was selected because the study was designed to evaluate DNA damage in relation to chemotherapy response and remodeling of the tumor–immune microenvironment, for which damage within tissue immediately surrounding the malignant epithelium may also be biologically relevant. Consequently, RADD scores represent tissue-level lesion burden within the tumor-containing microenvironment and should not be interpreted as measurements restricted exclusively to malignant epithelial cells.

Assay conditions were optimized and controlled for each antibody, fluorescence detection channel and antigen retrieval condition. Reference tissues were analyzed to control for batch-to-batch and intensity variability [12]. Staining and batch-control slides were processed alongside study specimens to evaluate signal specificity, background fluorescence, and consistency across experimental runs. Image acquisition settings, including objective, exposure parameters, and fluorescent channel settings, were held constant across samples to permit direct comparison of fluorescent intensity

### 2.4. NanoString Gene Expression Analysis

Total RNA was extracted from FFPE sections using PureLink^TM^ FFPE Total RNA Isolation Kit (Invitrogen, Waltham, MA, USA), cleaned and concentrated with the RNA Clean and Concentrator^TM^-5 (Zymo Research, Irvine, CA, USA) and quantitated by NanoDrop spectrophotometry (DeNovix, Wilmington, DE, USA). Gene expression profiling was performed on 23 pre-NACT and 20 post-NACT samples with total RNA (250 ng) using NanoString nCounter PanCancer I/O 360 Panel (Bruker Spatial Biology, Seattle, WA, USA), interrogating 770 genes encompassing immune cell types, immune pathways, tumor signatures, and metabolic processes.

The raw data from the NanoString panel were further evaluated on the ROSALIND platform (ROSALIND, San Diego, CA, USA) based on the nSolver 4.0 and Advanced Analysis Software 2.0 to assess sample similarity and volcano plots to depict differential gene expression comparing high versus low oxRADD tumors with expression normalized to a panel of 20 housekeeping genes employing unpaired *t*-tests with Benjamini–Yekutieli false discovery rate correction for multiple testing.

Cell type abundance scores were calculated from predefined cell type-specific gene sets using mean log_2_-transformed expression values with quality-controlled markers exhibiting high inter-gene correlation [13]. Pathway scores were derived from first principal component analysis (PCA) of constituent gene expression values within each pathway.

### 2.5. Statistical Analysis

Continuous variables were described as median or mean (standard error of the mean, SEM) depending on the analysis performed. Comparison between groups was performed using Welch’s *t*-test. Receiver operating characteristic (ROC) curve analysis was performed to evaluate the predictive performance of oxRADD for recurrence. The area under the curve (AUC) with 95% confidence intervals (CI) was calculated to quantify discriminative ability. The optimal cutoff for oxRADD score was determined from the ROC using the Youden index, defined as the threshold maximizing the sum of sensitivity and specificity minus one. Prediction for recurrence was evaluated using a penalized multivariate logistic regression model with a Firth correction. This approach was selected to reduce sample bias and improve coefficient stability with the limited number of observations (*N* = 38) and covariates tested. Continuous variables were entered as continuous terms, and categorical variables were coded according to the original dataset definitions. Penalized estimates were reported as odds ratios (ORs) with corresponding confidence intervals and *p*-values.

Survival outcomes were analyzed using Kaplan–Meier curves, and comparisons between groups were performed using the log-rank test. All tests were two-tailed, and *p* < 0.05 was considered statistically significant. For gene expression analysis, Benjamini–Yekutieli (BY) adjustment was applied. For gene signatures, multiple *t*-tests with a two-stage step-up Benjamini, Krieger, and Yekutieli correction and a false discovery rate (FDR) of 0.05 were performed. Analyses were performed using GraphPad Prism version 10.0, R version 4.2.1, and nSolver Analysis Software version 4.0.

## 3. Results

### 3.1. Patient Characteristics and DNA Damage Distribution

We retrieved 40 matched pre-NACT biopsies and post-NACT IDS specimens from patients treated at UAB from 2014 to 2022. Of the retrieved samples, further pathological analysis of IDS samples determined that one tumor was low-grade serous carcinoma, one tumor was clear cell carcinoma, and one tumor was endometrioid carcinoma; the remaining 37 were HGSOC. Given the epithelial origin and the similarities in first-line treatments and outcomes between endometrioid and HGSOC, we analyzed the 38 matched pairs of DNA damage.

All patients had stage III–IV disease, and all patients received platinum-based NACT consisting of 3–6 cycles of a carboplatin and taxane-based regimen. One patient received a total of 7 cycles due to the initial cycle being a carboplatin-only lead-in. Median age at diagnosis was 65.5 years (range 58–69 years). The majority of patients had an optimal interval cytoreduction (92.1%). At the time of analysis, 31 patients (81.5%) experienced disease recurrence, and 28 (73.4%) were deceased with a minimum 12-month follow-up (Table 1).

**Figure 1:**
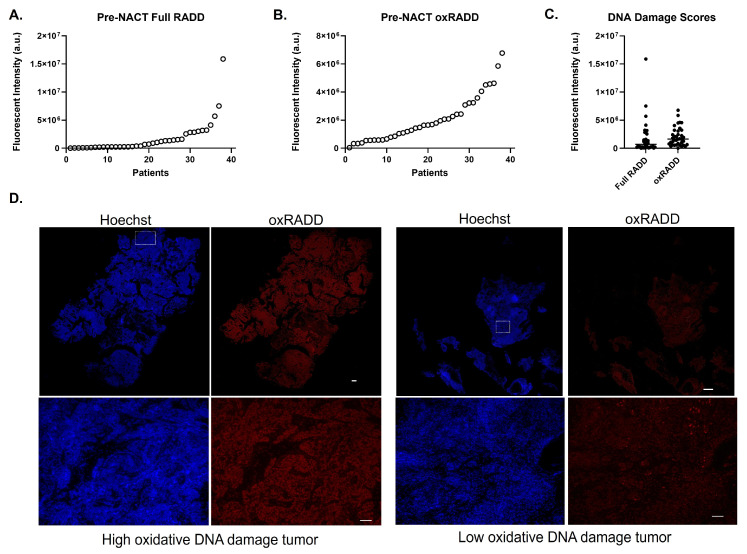
RADD DNA damage analysis of pre- and post-neoadjuvant (NACT) epithelial ovarian tumor samples. (**A**) Damage score distribution for chemonaïve pre-NACT tumor sections with the broad-spectrum Full RADD, which detected oxidative, crosslinked, and alkylated lesions along with uracil and abasic sites. (**B**) Oxidative damage (oxRADD) score distribution. (**C**) Comparison of Full RADD and oxRADD scores from each patient in the cohort. Dots reflect individual tumors, and the mean ± standard error of the mean (SEM) is shown. (**D**) Representative images of a high and low oxRADD tumor from the pre-NACT samples. The top is the stitched whole tumor image, and the bottom is an inset of the tumor. The inset is indicated on the Hoechst image with the white box. Scale bar for stitched image is 500 µm and 100 µm for the inset.

Using adjacent tissue sections, we performed a broad-spectrum analysis of DNA lesions (Full RADD) and a specific analysis for oxidative DNA lesions (oxRADD) within the pre- and post-NACT tumor tissue (Figure 1). The Full RADD cocktail detects abasic sites, alkylated damage, deamination events, DNA crosslinks, oxidative DNA damage, and strand breaks [10,14]. The oxRADD cocktail is specific for oxidative lesions and uses DNA repair enzymes formamidopyrimidine DNA glycosylase (FPG), endonuclease VIII (EndoVIII), and endonuclease IV (EndoIV) to remove oxidative DNA adducts for fluorescent tagging [9]. The mean fluorescent intensity of the tumor tissues for each damage cocktail was determined with the distribution of the broad spectrum of DNA damage using Full RADD and oxidative DNA damage using oxRADD, as shown in Figure 1A,B. Pre-NACT Full RADD scores had a median fluorescence intensity of 7.21 × 10^5^ (2.50 × 10^5^ to 1.53 × 10^6^ arbitrary units (a.u.)). oxRADD scores had a median of 1.64 × 10^6^ (4.55 × 10^4^ to 6.78 × 10^6^) (Figure 1C). The elevation in oxRADD scores relative to Full RADD scores is consistent with our previous analysis of 206 HGSOC tumors, in which we also observed a significant contribution of oxidative DNA damage to the overall damage spectrum, with oxRADD scores elevated relative to Full RADD [9]. The enzyme cocktail works by removing the damaged bases from the genome before gap filling occurs. If the DNA becomes too fragmented, Klenow polymerase efficiency drops, and labeling with the modified dUTP decreases, reducing fluorescence intensity. Similarly, if DNA damage is closely spaced, the excised fragment becomes much larger, creating a single-strand break rather than individual lesion sites, potentially reducing labeling and subsequent fluorescence intensity [15,16]. High levels of damage may result in lower Full RADD scores due to the higher number of glycosylase enzymes used in that cocktail. Therefore, we focused our analysis on the oxidative lesion content, given the significant contribution to the overall score.

### 3.2. Oxidative DNA Damage Associates with Recurrence Risk

**Figure 2:**
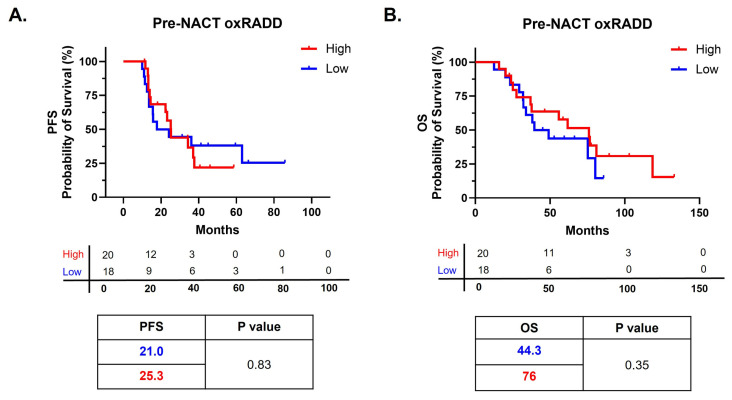
Median oxRADD determined high and low oxidative DNA damage status in the chemonaïve pre-NACT tumor tissues. (**A**) Progression-free survival (PFS) was not significantly different between groups, but the low damage tumors showed a slight delay in progression. (**B**) Overall survival (OS) was also not significantly different, but the high damage tumors showed a longer overall survival time.

We first examined the ability of oxRADD scores to predict progression-free survival (PFS) and overall survival (OS) (Figure 2). We determined the median oxRADD score and classified values above the median as high oxRADD (high oxidative DNA damage) and values below the median as low oxRADD (low oxidative DNA damage). Using the high and low oxRADD categories, we then examined the relationship between DNA damage levels and survival endpoints. Levels of oxidative DNA damage were not predictive of either PFS or OS. Tumors with high oxRADD prior to NACT showed a clinically meaningful, though not statistically significant, higher median OS (76 vs. 44.3 months, *p* = 0.35), suggesting that oxidative DNA damage influences tumor characteristics (Figure 2B).

**Figure 3:**
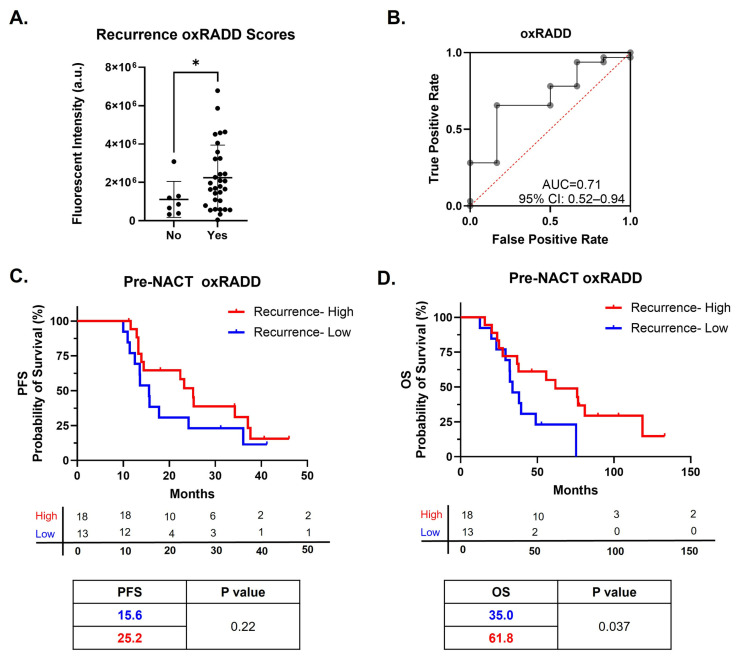
oxRADD scores are significantly higher in tumors that recur. (**A**) Mean fluorescent oxRADD values for pre-NACT tumors that are recurrent (*n* = 31) versus non-recurrent (*n* = 7, *p* = 0.028). (**B**) ROC analysis of oxRADD predicting recurrence after NACT. AUC values with 95% confidence intervals are shown. (**C**) PFS was not different between recurrent patients. (**D**) OS was significantly different, with serious damage to recurrent tumors showing a longer overall survival time. * *p* < 0.05.

We then examined the association between oxidative DNA damage levels before NACT and recurrence. Within 48 months, a majority of patients in the cohort had disease recurrence after initial treatment with IDS and chemotherapy (*n* = 31). Tumors that would go on to recur demonstrated elevated pre-NACT oxRADD scores compared to non-recurrent cases (2.24 × 10^6^ vs. 1.11 × 10^6^, *p* = 0.028; Figure 3A). Given the elevated levels of oxidative DNA lesions in recurrent samples, we tested the oxRADD score for recurrence prediction. We used a receiver operating characteristic curve (ROC) to examine predictive performance, and a Youden-derived cutoff of 1.43 × 10^6^ was chosen post hoc *(*Figure 3B; AUC = 0.71, 95% CI 0.52–0.94, cutoff). The resulting analysis provided a sensitivity of 0.6562, a specificity of 0.8333, a positive predictive value (PPV) of 0.9545, and a negative predictive value (NPV) of 0.3125, demonstrating a meaningful discrimination of chemonaïve tumors with respect to future recurrence. Given the small sample size and the data-derived cutoff, these results are exploratory, but the data suggest that oxidative DNA damage levels provide insight into recurrence risk.

Kaplan–Meier analysis of patients who would go on to recur, stratified by median oxRADD, revealed no significant differences in PFS (Figure 3C). However, patients with high oxRADD scores pre-NACT exhibited significantly improved OS compared to low oxRADD scores (61.8 vs. 35.4 months from diagnosis, hazard ratio [HR] = 0.42, 95% CI 0.17–1.03, *p* = 0.037; Figure 3D).

### 3.3. Platinum Sensitivity Correlates Inversely with oxRADD in Recurrent Disease

**Figure 4:**
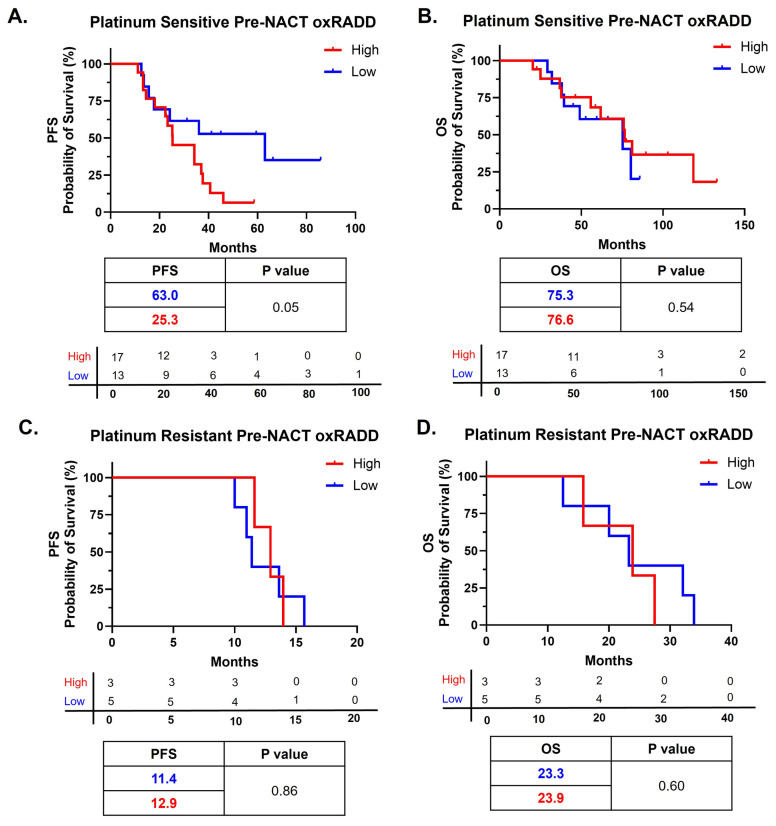
oxRADD scores were predictive of PFS for platinum-sensitive tumors. (**A**) PFS was significantly different between platinum-sensitive low (*n* = 15) and high (*n* = 15) DNA damage patients. (**B**) OS was not significantly different. (**C**) PFS was not different between platinum-resistant low (*n* = 3) and high (*n* = 5) DNA damage patients. (**D**) OS was not significantly different for platinum-resistant tumors.

We then examined the association with platinum status. In this cohort, 30 patients were platinum-sensitive, and eight were platinum-resistant. Platinum sensitivity was defined as a platinum-free interval (PFI) of 6 months or greater after completion of primary platinum-based chemotherapy, and platinum resistance was defined as recurrence within 6 months of completion of primary platinum treatment. There were no platinum refractory patients in this study. Among platinum-sensitive patients (*n* = 30), low pre-NACT oxRADD scores predicted significantly improved PFS compared to high oxRADD scores (63.0 vs. 25.3 months, HR = 0.43, 95% CI 0.19–1.0, *p* = 0.05; Figure 4A). OS did not show a significant association with oxRADD scores, and the survival time was similar between the two groups (75.3 vs. 76.6 months, *p* = 0.54; Figure 4B). Platinum-resistant patients showed no difference in PFS (11.4 vs. 12.9 months, *p* = 0.86, Figure 4C) or OS (23.3 vs. 23.9 months, *p* = 0.60; Figure 4D) based on oxidative DNA damage. This trend of improved OS in the platinum-resistant, low-damage group was more consistent with the PFS observed for the platinum-sensitive patients, the recurrent patients, and the entire cohort, where low oxidative DNA damage conferred a survival advantage compared to the high oxidative DNA damage patients, though the significance varied by category. Interestingly, the OS trends for the platinum-sensitive patients, the recurrent patients, and the entire cohort tended to have no difference or slightly favor the high oxidative DNA damage tumors, suggesting some tumor remodeling or another factor is influencing the overall survival. The statistical analysis of the resistant group is limited by the small number of patients, but the trend is interesting.

### 3.4. HRP Tumors Harbored Greater Oxidative DNA Damage

**Figure 5:**
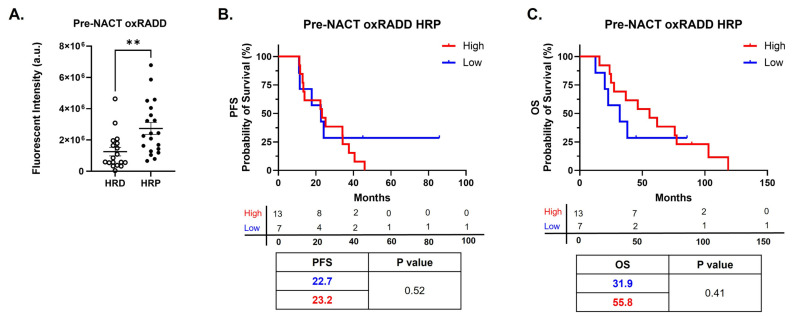
oxRADD scores were significantly elevated in HRP tumors, though survival outcomes between groups were not changed. (**A**) Mean fluorescent oxRADD values for HRP (*n* = 20) and HRD (*n* = 18) with status determined by Caris Life Science’s MI profiling or Foundation One CDx assay (*p* = 0.0034). Dots are individual tumors, and the mean ± SEM is shown. Significance was determined by a Welch’s *t*-test, with *p* < 0.01 (**). (**B**) PFS was not different between HRP low (*n* = 10) and high (*n* = 10) DNA damage patients. (**C**) OS was also not significantly different for HRP tumors.

While assessing the 92 patients enrolled in the VITAL trial, we discovered that HRP tumors were associated with higher Full RADD scores [11]. Therefore, we examined the relationship between homologous recombination status and oxidative DNA damage in this cohort. Of the available 38 patients evaluable for oxRADD, 52.6% (20/38) were deemed HRP after chart review. Homologous recombination status was determined for all patients using either Caris Life Science’s MI profiling or Foundation One CDx assay, depending on institutional preference at the initial diagnosis time point, plus germline testing. Oxidative DNA damage was significantly associated with HRP status, with mean oxRADD scores among HRP tumors more than double those of HRD tumors (2.74 × 10^6^ vs. 1.25 × 10^6^, *p* = 0.0034; Figure 5A). The Full RADD scores also confirmed HRP status (2.38 × 10^6^ vs. 1.25 × 10^6^, *p* = 0.016). Amongst HRP patients who did not experience recurrence, there was significantly lower oxRADD compared to patients who did recur (1.04 × 10^6^ vs. 3.03 × 10^6^, *p* = 0.0004); however, the number of HRP patients without recurrence was very low (*n* = 3). Given that oxRADD is elevated in HRP tumors, which traditionally have lower chemosensitivity and worse survival, we evaluated survival outcomes in the HRP group, stratified by median oxRADD. The HRP group did not show improved PFS (23.2 vs. 22.7 months, *p* = 0.52; Figure 5B) or OS (55.8 vs. 31.9 months, *p* = 0.41; Figure 5C) when stratified by oxidative DNA damage. Again, the OS shows an improved survival time, though not significant, for the high oxidative lesion-containing group, providing more evidence that another tumor intrinsic factor is contributing to survival and oxidative lesions.

### 3.5. Oxidative DNA Damage and Clinical Markers Combine to Improve the Prediction of Recurrence

Given the associations of oxidative DNA damage with recurrence, platinum sensitivity, and homologous recombination status, we conducted a multivariate analysis to examine predictors for recurrence, examining oxRADD, primary platinum status, number of NACT cycles, large ascites, and homologous recombination status. Given the small sample size, we used a Firth-style penalized multivariate model to reduce bias and improve separation given the limited set of observations. In this penalized model (Table 2), oxRADD remained independently associated with recurrence after adjustment for the additional variables. High oxRADD was associated with increased odds of recurrence (odds ratio (OR) 1.27, 95% CI 1.16–1.39, *p* < 0.0001). Among the covariates, primary platinum status was also significant (OR 1.21, 95% CI 1.07–1.36, *p* = 0.002), as were NACT cycles (OR 1.18, 95% CI 1.04–1.234, *p* = 0.011), large ascites (OR 1.15, 95% CI 1.04–1.28, *p* = 0.008), and homologous recombination status (OR 1.15, 95% CI 1.02–1.29, *p* = 0.022). Age and chemo regimen were not independently associated with recurrence in the adjusted model. While the model showed good discrimination, it is considered exploratory due to the small sample size and the need for a penalized model.

### 3.6. Neoadjuvant Chemotherapy Increases Oxidative DNA Damage

**Figure 6:**
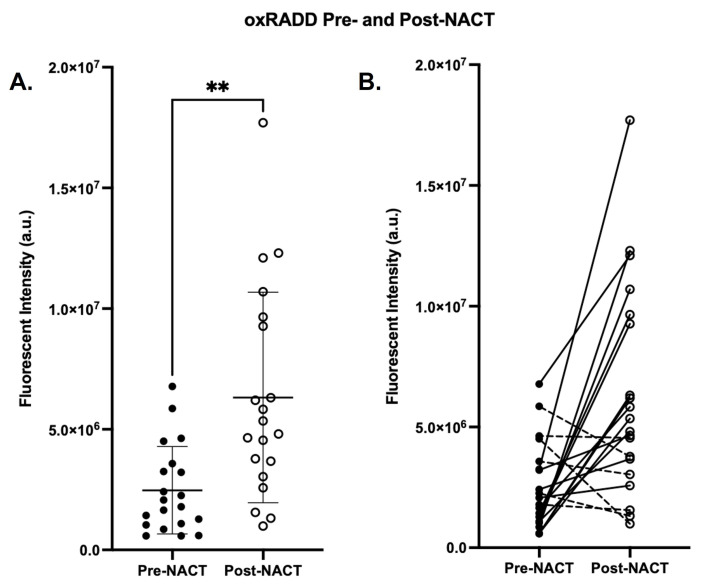
(**A**) Mean fluorescent oxRADD values for matched pre-NACT and post-NACT tumor specimens (*n* = 20, *p* = 0.002 (**)). (**B**) Paired oxRADD scores for individual patients from pre- to post-NACT. Solid lines indicate patients with increased oxRADD post-NACT (*n* = 14); dashed lines indicate patients with decreased oxRADD post-NACT (*n* = 6).

To determine whether platinum-based NACT alters the oxidative lesion landscape established at baseline, we compared oxRADD scores in matched pre- and post-NACT tumor specimens. A total of 20 out of 38 patients had sufficient tumor for evaluation with oxRADD at IDS; therefore, we used the oxRADD scores of these 20 patients for paired comparison before and after NACT. In these pairs, mean oxRADD scores were significantly elevated from pre- to post-NACT (2.47 × 10^6^ vs. 6.32 × 10^6^, *p* = 0.002; Figure 6A), and oxRADD in 14 of 20 tumors (70%) increased from pre-to post-NACT analysis (Figure 6B). Among the six patients whose oxRADD decreased post-NACT, all had pre-NACT oxRADD scores above the cohort median. The persistence and chemotherapy-induced increase in oxidative lesion burden supported continued investigation into whether pre-NACT oxidative DNA damage status was associated with transcriptomic differences in the tumor–immune microenvironment.

### 3.7. Oxidative DNA Damage Alters the Immune Microenvironment

In our previous oxRADD studies using tumor microarrays, we noted a spatial correlation between oxidative DNA damage regions and PD-L1 expression [9]. The tumor cores in that study were primarily from Asian countries and lacked clinical data for other correlations. When we examined the broad spectrum of DNA damage in the VITAL trial (NCT02346747), we observed alterations in the immunotranscriptomics of the chemonaïve tumors, which showed that high DNA damage levels attenuated the immune microenvironment and were associated with an improved response to the autologous tumor cell immunotherapy Vigil (gemogenovatucel-T) [11]. Given that high levels of oxidative DNA damage were associated with recurrence and improved OS for recurrent patients, we performed the same gene expression profiling on 23 pre-NACT and 20 post-NACT samples using the NanoString PanCancer I/O 360 panel. We were limited by the available tissue for this analysis, given that the pre-NACT samples came from biopsies with limited available tissue, some of which were exhausted during initial diagnostic sectioning or subsequent translational analyses, and not all post-NACT samples contained tumor tissue sufficient for evaluation due to initial chemotherapy response.

**Figure 7:**
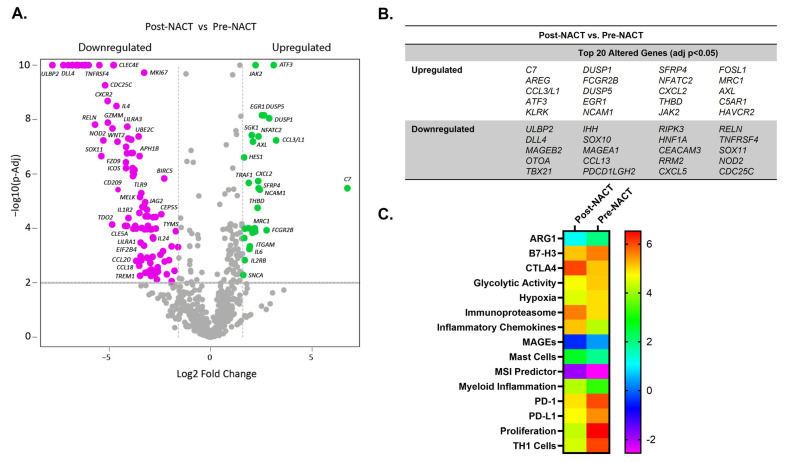
(**A**) Volcano plot of the differential expression of genes post-NACT relative to pre-NACT exposure in the tumor sample, measured by NanoString PanCancer I/O 360. Genes in violet indicate downregulated genes with a Benjamini–Yekutieli (BY) adjusted *p*-value < 0.05; green indicates upregulated genes with a Benjamini–Yekutieli (BY) adjusted *p*-value < 0.05. (**B**) Table of the top 20 up- and downregulated genes. (**C**) Heat map of the differential expression of NanoString signatures between the pre- and post-NACT samples. The mean score of signatures with adjusted *p* < 0.05 determined by an unpaired *t*-test with FDR 0.05 is shown.

We first compared the pre- and post-NACT transcriptomics to understand the NACT response. In total, 82 genes from this panel were significantly upregulated (fold change > 2 and adj *p* < 0.05) after chemotherapy exposure, and 152 genes were significantly downregulated (fold change < −2 and adj *p* < 0.05) (Figure 7A). The top upregulated and downregulated genes (Figure 7B) are consistent with prior transcriptomic analyses comparing pre- and post-NACT tissues [4].

We then examined the changes in immune signatures determined by the panel (Figure 7C). We observed a significant downregulation of immune signatures in the post-NACT samples associated with anti-tumor T cells and glycolytic activity. We also observed an upregulation of signatures associated with the immunoproteasome, antigen generation for antigen-presenting cells (APCs), stimulation of helper T cells (Th1 cells), antigen-presenting machinery (APM), inflammatory chemokines and myeloid inflammation signatures post-NACT. Along with pro-inflammatory TIME signatures, there was an increase in signatures representing infiltration of CD8+ T cells, macrophages, and mast cells. Additionally, general cytotoxic cell signatures were also increased, though not statistically significant (*p* = 0.07). The increase in inflammatory markers and immune cell infiltration signatures was observed despite no significant increase in myeloid cells and downregulation of the major, highly cytotoxic subset of natural killer cells (CD56^dim^ NK cells), Th1 cells, and dendritic cells, suggesting a more efficient anti-tumor–immune microenvironment with the loss of immunosuppressive mechanisms (Figure 7C).

We next examined transcriptomic differences between high and low oxRADD cohorts within the pre-NACT and post-NACT groups separately. oxRADD status was determined using the chemonaïve oxRADD score.

**Figure 8:**
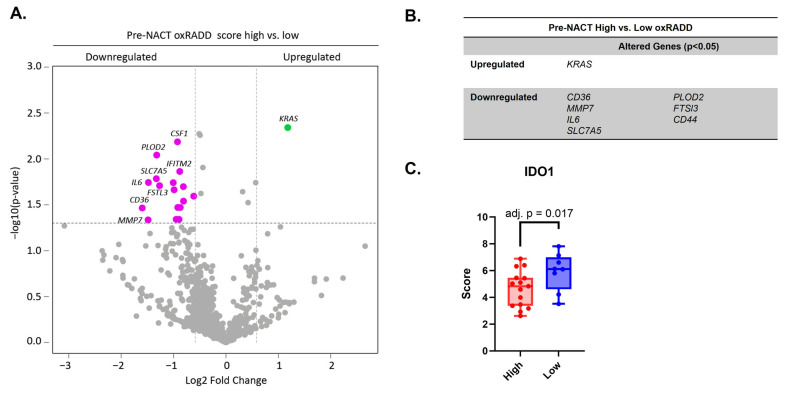
(**A**) Volcano plot of differential expression of genes in pre-NACT tumors stratified by high or low pre-NACT oxRADD; upregulated (green) and downregulated (violet) genes with *p* < 0.05. (**B**) List of all genes with differential expression with *p*-value < 0.05 and fold-change > 2. (**C**) The NanoString IDO1 expression signature showed significant differences between groups before chemotherapy. The box plot shows the mean score for the signature, with the min and max ranges indicated, and the significance determined by an unpaired *t*-test with FDR 0.05.

Within the pre-NACT group, no genes were significantly upregulated or downregulated by adjusted *p*-value, likely due to the small sample size. When using standard *p*-value for significance, *KRAS*, associated with tumorigenesis, chemoresistance, and progression, was the only gene significantly upregulated in the high oxRADD cohort (fold change 2.27, *p* = 0.005). Seven genes were noted to be downregulated with fold change > 2 and standard *p*-value < 0.05 (Figure 8A,B), impacting the hypoxia inducible factor (HIF)/hypoxia signaling pathway, the TGF-β/SMAD signaling pathway, and multiple immune signaling pathways [17,18,19,20,21,22]. Analysis of all NanoString immune signatures together showed significant changes in Indoleamine 2,3-dioxygenase 1 (IDO1), a negative regulator of tumor immunity (adjusted *p* = 0.017, Figure 8C). Examining the individual signatures, TGF-β activity (*p* = 0.013) and myeloid cells (*p* = 0.045) also showed decreased activity in the high oxRADD group.

**Figure 9:**
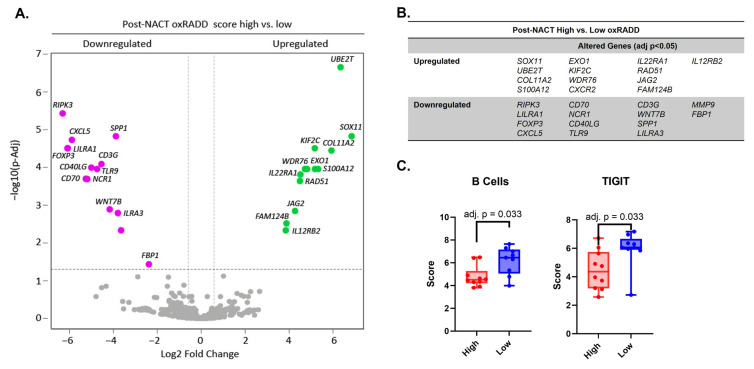
(**A**) Volcano plot of differential expression of genes in post-NACT tumors stratified by high or low pre-NACT oxRADD; upregulated (green) and downregulated (violet) genes with adj *p* < 0.05. (**B**) List of all genes with differential expression with a Benjamini–Yekutieli (BY) adj *p*-value < 0.05 and fold-change > 2. (**C**) The box plot shows the mean score for the signatures with significant changes. The min and max ranges are indicated, and the significance was determined by an unpaired *t*-test with FDR 0.05.

Among the post-NACT group, differences by pre-NACT oxRADD status were more pronounced. Figure 9 shows the significantly increased and decreased genes from post-NACT tumor transcriptomics with patients stratified by pre-NACT oxRADD scores, and the magnitude of fold change therein. The B cell NanoString signature was significantly increased in the low oxidative DNA damage tumors after chemotherapy exposure. Immune modulatory, T cell-associated TIGIT signature was also significantly elevated in the low oxRADD cohort (Figure 9C). We compared the TGF-β activity signatures in the pre- and post-NACT samples and observed a significant decrease for both high oxRADD groups compared to their respective low oxRADD groups (*p* = 0.021) across this signature.

## 4. Discussion

Two key findings from this study were that pre-NACT oxidative DNA lesions, measured via oxRADD, were predictive for recurrence and HRP status. oxRADD significantly predicted recurrence in HGSOC (*p* = 0.028, AUC = 0.71). Recurrent tumors harbored nearly three-fold higher oxRADD scores than non-recurrent tumors, suggesting oxidative DNA damage as a measurable pretreatment biomarker for disease recurrence. This is consistent with the broader understanding that oxidative stress promotes genomic instability, proliferative signaling, and immune evasion in ovarian cancer [23,24].

Interestingly, among patients who did recur, high oxidative DNA damage scores were paradoxically associated with improved overall survival (61.8 vs. 35 months, HR = 0.42, *p* = 0.037). This finding that oxRADD scores may predict both higher recurrence risk and better post-recurrence survival suggests that oxidative DNA damage captures biologically distinct tumor properties across different clinical time points. We have seen in prior tissue analysis comparing platinum-sensitive to platinum-refractory tumors that platinum-sensitive tumors harbored higher oxidative DNA damage, suggesting a possible link to chemosensitivity through genomic instability [25]. Therefore, one interpretation is that although recurrence risk may be greater in these patients due to innate tumor characteristics, they have an improved responsiveness while actively being treated with chemotherapy. Another interpretation is that tumors with high oxidative damage harbor an increased neoantigen burden or altered immune microenvironments, rendering them more susceptible to subsequent therapies over time [11]. This second scenario is supported by the VITAL trial data, where elevated Full RADD (all lesion types) scores predicted improved response to Vigil immunotherapy, and by the established literature linking genomic instability to inflammatory signaling that enhances anti-tumor immunity [26].

Similarly, HRP tumors exhibited more than double the oxRADD scores of HRD tumors (2.74 × 10^6^ vs. 1.25 × 10^6^, *p* = 0.0034), confirming observations of DNA damage correlation with homologous recombination status from the VITAL trial [11]. The strong association between oxidative lesions and homologous recombination status is consistent with emerging evidence linking homologous recombination status to oxidative metabolism. Farmanbar et al. demonstrated an inverse relationship between HRD and ROS-associated mutational signatures in breast cancer, where ROS signatures were uniquely more present in HRP tumors and absent in HRD tumors [27]. HRD cancers are predicted to rely on oxidative phosphorylation for PARP-dependent repair rather than glycolysis, causing increased oxidative stress to slow replication dealt with by compensatory antioxidant mechanisms [28,29]. Therefore, HRP tumors may have oxidative lesions due to their differences in metabolism and redox status. Further, RADD measures persistent DNA lesions that remain unrepaired within the genomic DNA at the time of analysis. Elevated error-prone repair mechanisms, which increase mutation burden, would repair, though incorrectly, the DNA lesions, reducing the RADD signal. Therefore, the unique state of the tumor and surrounding tissue is reflected by the lesion load and detected by both the Full RADD and oxRADD.

The association between lower oxidative DNA damage and HRD tumors may also explain the paradoxical survival curves seen in the platinum-sensitive patients, where the lower oxidative damage group is being anchored by HRD patients with disproportionately strong chemoresponsiveness. The three patients within the HRP group that had not recurred harbored a mean oxRADD score similar to the HRD group (1.04 × 10^6^ and 1.25 × 10^6^, respectively), suggesting a possible “HRD-ness” factor contributing to lower oxidative damage that is not explained through oxRADD alone. Conclusions are limited by the small sample size, but the continued association between oxRADD and homologous recombinationstatus is hypothesis-generating and warrants validation in larger cohorts.

oxRADD scores also highlight a unique relationship between oxidative lesion levels and platinum sensitivity. Low pre-NACT oxRADD scores were associated with improved PFS in platinum-sensitive patients (63.0 vs. 25.3 months for low vs. high oxRADD, *p* = 0.05). This difference could be explained by anchoring from HRD patients with lower oxidative DNA damage, as previously discussed, or low oxRADD scores may reflect more efficient DNA repair capacity, resulting in less persistent DNA damage, which confers longer remissions following platinum-based chemotherapy. The absence of significant oxRADD-associated survival changes in the platinum-resistant patients (*n* = 8) limits the interpretation in that subgroup. Further investigation is needed to elucidate biological differences in genomic stability among platinum-sensitive, platinum-resistant, and even refractory tumors, with particular interest in intrinsic “HRD-like” factors that contribute to the responsiveness of tumors with low oxidative lesions that are platinum-sensitive.

In addition to its predictive value at baseline, oxRADD captured dynamic changes during treatment. NACT significantly increased oxidative DNA damage in matched tumor specimens (2.47 × 10^6^ vs. 6.32 × 10^6^, *p* = 0.002), consistent with the known mechanism of platinum agents, which accumulate in mitochondria and generate reactive oxygen species as a component of their cytotoxicity [30]. Notably, the six patients whose oxRADD decreased post-NACT all had pre-NACT oxRADD scores above the cohort median, suggesting that tumors with already-elevated oxidative lesion burdens may engage compensatory antioxidant or repair responses during chemotherapy exposure. This pattern may also reflect selection pressure, where tumor cells with the most serious baseline oxidative damage are preferentially eliminated by NACT, leaving behind clones with lower oxidative lesion loads. Whether this decrease reflects adaptive resistance mechanisms or clonal selection warrants further investigation, as it may inform a predictive model of platinum responsiveness in patients whose tumors exhibit decreased oxidative damage following chemotherapy.

Given the associations observed between oxidative DNA damage, recurrence, platinum response, and survival, we used a limited multivariate model to examine whether oxRADD and other clinical variables predicted recurrence. Using a penalized model, we observed significant associations between oxRADD, primary platinum status, NACT cycles, ascites volume, and homologous recombination status (Table 2). While the analysis is strongly limited by the small sample size, it supports oxidative DNA damage playing a role in shaping therapeutic response.

The influence of DNA damage on the tumor and its microenvironment was further supported by our transcriptomic analysis. Stratifying by oxRADD status, we observed that high oxidative DNA damage is associated with a distinct immune phenotype characterized by lower TGF-β activity, reduced myeloid cell infiltration, and decreased IDO1 expression in the chemonaïve TIME. TGF-β was also associated with oxRADD status in pre- and post-NACT analyses, remaining significantly lower in high oxRADD tumors at both timepoints. Given the well-established role of TGF-β in mediating T-cell exclusion, promoting stromal activation, and suppressing antigen presentation in ovarian cancer [31,32,33]. The association between high oxidative damage and lower TGF-β signaling may partially explain the paradoxical survival benefit observed in the overall survival for recurrent and platinum-sensitive patients with high oxRADD. TGF-β reduces MHC-1 expression and activates fibroblasts to produce barriers in the extracellular matrix, preventing T cells from infiltrating the TIME, and the neutralization of TGF-β has been shown to improve anti-tumor–immune response and reduce progression in murine models [34]. Therefore, the lower TGF-β environment in high oxRADD tumors suggests less immunosuppression after therapy, permitting more effective immune surveillance despite the higher recurrence rate.

In high oxRADD pre-NACT tumors, the upregulation of *KRAS* and the downregulation of genes in the HIF/hypoxia and TGF-β/SMAD pathways (*CD36*, *MMP7*, *IL6*, *SLC7A5*, *PLOD2*, *FSTL3*, *CD44*) further support a model in which high oxidative-damage tumors exhibit reduced hypoxic and immunosuppressive signaling. Post-NACT, high oxRADD tumors showed elevated hypermutation and APM loss signatures, along with upregulation of specific DNA repair genes (*RAD51*, *EXO1*, *UBE2T*, *KIF2C*, and *WDR76*), suggesting active genomic instability and compensatory repair responses. Conversely, low oxRADD tumors post-NACT demonstrated greater B cell infiltration. Along with elevated TIGIT expression in low oxRADD tumors, suggesting a more immune-engaged but potentially exhausted TIME may further contextualize the paradoxical survival findings observed in Figure 3D and Figure 4A.

NACT with a platinum–taxane backbone modulates the tumor microenvironment through mechanisms beyond direct cytotoxicity, shifting the innate immunosuppressive characteristic of ovarian cancer toward one with reduced immune regulation and increased tumor recognition [35]. Our transcriptomic analysis confirms this paradigm, as signatures for multiple negative regulators of anti-tumor immunity, ARG1, B7-H3, PD-1, and PD-L1, are significantly downregulated following NACT, while immunoproteasome and antigen-presenting machinery signatures are upregulated. These findings are consistent with the prior NanoString-based analysis by James et al., who similarly identified upregulation of stress-response transcription factors *ATF3* and *EGR1* alongside immune infiltrative changes after treatment with platinum–taxane-based NACT in HGSOC patients [4]. The concurrent decrease in glycolytic activity signatures in the post-NACT group suggests a metabolic shift that may further support cytotoxic immune cell function by allowing for glucose availability necessary for effector T-cell activity within the TIME [36]. Notably, the increase in CD8+ T cell, macrophage, and mast cell signatures occurred despite decreases in CD56dim NK cell, Th1, and dendritic cell signatures, suggesting qualitative remodeling of immune composition rather than a uniform increase across all immune populations. This pattern is consistent with the three tumor-infiltrating lymphocyte response patterns described by Lo et al., where NACT augments pre-existing TIL responses but does not relieve all immunosuppressive mechanisms [37].

This study supports oxRADD as a potential biomarker that integrates DNA repair capacity with immune microenvironment characteristics in HGSOC. However, several limitations should be acknowledged. The sample size of 38 patients limits statistical power, particularly for subgroup analysis such as platinum-resistant (*n* = 8) and non-recurrent (*n* = 7) populations. The NanoString analysis suffered attrition due to tissue availability, with only 23 pre-NACT and 20 post-NACT samples evaluable. Homologous recombination status was determined through heterogeneous testing platforms (Caris or Foundation One CDx), which may introduce variability within an already small group. The retrospective nature and single-institution design may limit generalizability. Additionally, Full RADD and oxRADD were quantified across pathologist-confirmed tumor-containing tissue sections. Scores were representative of DNA damage content across stromal composition and immune cells within the tissue, consistent with previous analysis and enabling correlation with the bulk RNA analysis performed with NanoString [11]. Accordingly, Full and oxRADD should be interpreted as a tumor-level measure of DNA lesion burden including the TIME, rather than as a tumor-cell-specific measurement. The use of median oxRADD as the dichotomization threshold, while standard, may not represent the optimal biological cutoff for future use and requires validation in multiple independent cohorts. Despite these limitations, our transcriptomics findings confirm previous results using similar sample types and confirm the immune remodeling observed after NACT. This concordance supports DNA damage-specific analysis of immune remodeling and suggests that genomic instability and its intersection with the TIME needs further investigation with larger, multi-institutional cohorts to determine optimal oxRADD thresholds for clinical decision-making.

## 5. Conclusions

Oxidative DNA damage measured from chemonaïve biopsies predicted recurrence, associated with distinct immune phenotypes characterized by reduced TGF-β and IDO1 signaling, and correlated with improved overall survival in patients with recurrent disease. These findings, in the context of prior VITAL trial data demonstrating that DNA damage levels predict immunotherapy response, suggest that oxRADD may serve as a pre-treatment stratification tool for both prognostication and therapeutic selection. This is particularly important as immunotherapy has entered the National Comprehensive Cancer Network (NCCN) guidelines for treatment of recurrent ovarian cancer [38] and is currently undergoing investigational use as an addition to standard platinum–taxane doublet in the upfront setting [39,40]. As we look to optimize treatment with immunotherapeutic agents, oxRADD may offer actionable insight into tumors with less immunosuppressive TIMEs driven by high oxidative DNA damage.

## Figures and Tables

**Table 1:** Clinical characteristics of study cohort (*N* = 38 OC patients with matched pre- and post-NACT tissue).

Variable	*n*	Median (95% CI)
Age	38	65.50 (58.00–69.00)
BMI (kg/m^2^)	38	27.40 (25.40–31.53)
Race		
*White*	27	
*Black*	10	
*Asian*	1	
Chemo Regimen		
*carboplatin/paclitaxol*	24	
*carboplatin/taxotere*	9	
*carboplatin/dose-dense taxol*	5	
OS (Months)	38	47.72 (33.90–66.33)
Status		
*dead*	28	
*alive*	8	
*lost to follow-up*	2	
PFI (months)	38	15.30 (8.60–32.23)
PFS (months)	38	23.28 (14.17–37.30)
Recurrence		
*yes*	31	
*no*	7	
Kelim Score	31	1.10 (0.95–1.47)
Homologous Recombination Status		
*Proficient*	20	
*Deficient*	18	
Cytoreduction Status		
*R0*	25	
*R1*	10	
*R2*	3	
Number of NACT Cycles		
*3*	21	
*4*	11	
*6*	5	
*7*	1	
Co-morbidities		
*Yes*	29	
*No*	9	
FIGO Stage		
*IIIB*	1	
*IIIC*	29	
*IVA*	3	
*IVB*	5	
Primary Platinum Status		
*Platinum Resistant (PFI < 6 months)*	8	
*Platinum Sensitive (PFI > 6 months)*	30	

**Table 2:** Firth-style penalized multivariate model for predicting recurrence.

Variable	OR	95% CI	*p* Value
oxRADD	1.27	1.16–1.39	<0.0001
Age	0.96	0.83–1.11	0.596
Chemo Regimen	0.99	0.85–1.14	0.852
Primary Platinum Status	1.21	1.07–1.36	0.002
NACT Cycles	1.18	1.04–1.34	0.011
Large Ascites	1.15	1.04–1.28	0.008
Homologous recombination Status	1.15	1.02–1.29	0.022

## Data Availability

Dataset available in the Supplementary Materials. Images are available upon request from the authors.

## Supplementary Materials

The following supporting information can be downloaded at: https://www.mdpi.com/article/10.3390/cancers18152437/s1. The raw data of Table 1 and Figure 1, Figure 2, Figure 3, Figure 4, Figure 5, Figure 6, Figure 7, Figure 8 and Figure 9.

## Abbreviations

The following abbreviations are used in this manuscript:

HGSOC	High-grade serous ovarian cancer
NACT	neoadjuvant chemotherapy
RADD	Repair Assisted Damage Detection
oxRADD	Oxidative Repair Assisted Damage Detection
HR	Hazard ratio
AUC	Area under the curve
IDS	interval debulking surgery
ATF3	activating transcription factor 3
EGR1	early growth response 1
PFI	platinum-free interval
HRD	homologous recombination deficiency
HRP	homologous recombination proficiency
FFPE	formalin-fixed paraffin-embedded
FPG	formamidopyrimidine DNA glycosylase
FDR	False discovery rate
EndoVIII	endonuclease VIII
EndoIV	Endonuclease IV
AU	arbitrary unit
PFS	progression-free survival
OS	Overall survival
APCs	antigen-presenting cells
OR	Odds ratio
APM	antigen-presenting machinery
IDO1	Indoleamine 2,3-dioxygenase 1
NCCN	National Comprehensive Cancer Network
TIME	Tumor–immune microenvironment
